# NeuroEditor: a tool to edit and visualize neuronal morphologies

**DOI:** 10.3389/fnana.2024.1342762

**Published:** 2024-02-14

**Authors:** Ivan Velasco, Juan J. Garcia-Cantero, Juan P. Brito, Sofia Bayona, Luis Pastor, Susana Mata

**Affiliations:** ^1^Department of Computer Science, Universidad Rey Juan Carlos (URJC), Tulipan, Madrid, Spain; ^2^Center for Computational Simulation, Universidad Politecnica de Madrid, Madrid, Spain; ^3^DLSIIS, ETSIINF, Universidad Politecnica de Madrid, Madrid, Spain

**Keywords:** visualization, neuron morphology, tracing, neuron editing, correction, mesh, 3D, dendritic structure

## Abstract

The digital extraction of detailed neuronal morphologies from microscopy data is an essential step in the study of neurons. Ever since Cajal’s work, the acquisition and analysis of neuron anatomy has yielded invaluable insight into the nervous system, which has led to our present understanding of many structural and functional aspects of the brain and the nervous system, well beyond the anatomical perspective. Obtaining detailed anatomical data, though, is not a simple task. Despite recent progress, acquiring neuron details still involves using labor-intensive, error prone methods that facilitate the introduction of inaccuracies and mistakes. In consequence, getting reliable morphological tracings usually needs the completion of post-processing steps that require user intervention to ensure the extracted data accuracy. Within this framework, this paper presents NeuroEditor, a new software tool for visualization, editing and correction of previously reconstructed neuronal tracings. This tool has been developed specifically for alleviating the burden associated with the acquisition of detailed morphologies. NeuroEditor offers a set of algorithms that can automatically detect the presence of potential errors in tracings. The tool facilitates users to explore an error with a simple mouse click so that it can be corrected manually or, where applicable, automatically. In some cases, this tool can also propose a set of actions to automatically correct a particular type of error. Additionally, this tool allows users to visualize and compare the original and modified tracings, also providing a 3D mesh that approximates the neuronal membrane. The approximation of this mesh is computed and recomputed on-the-fly, reflecting any instantaneous changes during the tracing process. Moreover, NeuroEditor can be easily extended by users, who can program their own algorithms in Python and run them within the tool. Last, this paper includes an example showing how users can easily define a customized workflow by applying a sequence of editing operations. The edited morphology can then be stored, together with the corresponding 3D mesh that approximates the neuronal membrane.

## 1 Introduction

Understanding the multilevel structure and function of the brain is still one of the greatest challenges in science. One of the first steps along the path leading to this goal is necessarily understanding neurons, the basic building blocks of nervous systems.

Historically, the study of neuron anatomy has always provided fundamental insight into the nervous system, ever since the studies of Ramon Cajal (1888). Nowadays, new findings on neuron anatomy are still used in many research areas. For example, real and/or biologically-plausible neuron morphologies are incorporated soon after their acquisition to simulation studies, which seek the replication of the behavior of live neuronal circuits and allow performing analysis of procedures that are otherwise impossible to be implemented (Froeter et al., 2014; Beul and Hilgetag, 2019). Other studies also highlight the importance of neuron anatomy, which strongly affects, not only the physiology of the individual cells, but also the connectivity options inside neuronal circuits, given the impact that dendritic and axonal arbor morphologies have in the synapses’ conformation process (Halavi et al., 2008). Additionally, the morphological analysis of neurons was essential for categorizing neurons into different functional groups (Zeng and Sanes, 2017), and the study of neuron morphology has allowed the performance of comparative analyses among neurons acquired from different populations (DeFelipe et al., 2002), differentiated by multiple factors such as age (Duan et al., 2003; Kabaso et al., 2009), presence of mental disorders and their treatments (Mavroudis et al., 2021), etc. For example, anatomical studies have allowed characterizing the effects that certain disorders, such as Alzheimer, have over neuron morphology (Knafo et al., 2009).

Regarding the study of neuron anatomy, morphological tracings are useful for analysis operations, providing compact descriptions that have been traditionally used for data interchange within the neuroscientist community (Ascoli et al., 2007; Halavi et al., 2008; Akram, 2018). Furthermore, these descriptions facilitate performing morphological quantitative feature and data acquisition procedures, which helps studying neuron variability (Scorcioni, 2008; Bria, 2015). There are also some tools for neuronal volumetric model visualization (Abdellah, 2018) or neuronal volumetric images (Peng, 2014; Bria, 2015). However, obtaining good quality tracings is not always easy, as it is highly dependent on the quality of the microscopy images from which the tracings are acquired.

There are two main acquisition approaches: manual tracings over microscopy image stacks and the semi-automatic application of methods based on the automatic or supervised segmentation of these image stacks. Extracting traces manually is a labor intensive and time-consuming task, where the workload placed on human operators facilitates the occurrence of errors (Dercksen et al., 2013). Several attempts have been performed for obtaining these tracings automatically (Bas and Erdogmus, 2011; Chothani et al., 2011; Longair, 2011; Türetken et al., 2011; Wang et al., 2011; Zhao et al., 2011; Arshadi, 2021). However, so far none of them has yet reached results comparable to those of an experienced technician; additionally, the new methods introduce new problems (Donohue and Ascoli, 2011). As Peng et al. (2010) affirm, when tracings are automatically extracted, “sometimes searching for errors and correcting them may take a longer time than that needed for the manual tracing of the entire neuron.” Hence, no matter whether tracings are extracted manually or with some kind of automated procedure, there is always a need for tools that facilitate the process of finding and correcting errors.

There are different technological approaches for obtaining neuron tracings. In consequence, the complexity and heterogeneity of the tracings available to the scientific community is quite variable. But in any case, errors in the extracted tracings make subsequent analysis tasks more difficult, even producing erroneous analysis results (and this is true regardless of whether the tracings are extracted manually or (semi) automatically). In some cases, these errors can also prevent creating a mesh that approximates the membrane of the neuron described by that morphology tracing. Consequently, it can be stated that correcting tracing errors is an important step in the morphological analysis of neurons.

Commercial software such as Neurolucida (MicroBrightfield, VT, United States), Amira (Thermo Fisher Scientific, Massachusetts, United States) or Imaris and Filament tracer (Bitplane AG, Zurich, Switzerland) help in the reconstruction process from microscopic images and provide tools for neuron analysis. G-Tree (Zhou et al., 2009) is another tool to reconstruct a neuron from the stack of images where errors such as missing or misaligned neurites must be detected visually and can be corrected manually. A complete tool is the TREES toolbox (Cuntz et al., 2011) as it is open-source software in Matlab that allows visualizing a 3D polyline and prune branches, generating a cylinder structure from a real stack of images, and generate synthetic neurons. It offers statistical analysis tools and editing options. Other useful tools are neuTube (Feng et al., 2015), an open-source tool with capability of manipulating swc files that was designed for reconstructing neurons from a single tiff stack, and ShuTu (Jin et al., 2019), that overcomes the limitation of a single tiff stack and is specialized in the reconstruction of dendrites imaged using bright-field microscopy. SNT (Arshadi, 2021) and L-Measure (Scorcioni, 2008) are useful available tools designed to quantify neuronal anatomy.

This paper presents NeuroEditor, a tool for postprocessing, editing and visualizing reconstructed neuronal morphologies. The proposed methods facilitate extracting more accurate digital tracings by allowing correcting the errors introduced during the acquisition process. This, in turn, allows obtaining more precise measurements and features from the extracted tracings, or even carrying out computations that would be unfeasible if only uncorrected tracings were available. The methods presented here, also allow the inclusion of information not previously acquired or even the estimation of elements that may not be present in original acquired tracings, such as the soma description or the dendritic arbor thickness. This facilitates standardizing and comparing data acquired in different periods of time or coming from different laboratories or acquisition methods, for example, to create a database of cell models for simulation environments. Users can program their own methods in Python and use them directly in the tool.

Additionally, this tool allows generating geometric models of the neuronal membrane surface, reconstructed from the improved and homogenized tracings. These 3D models can be imported into standard 3D visualization tools to generate visualizations for dissemination or educational purposes, or they can be used in scientific studies involving the cell membrane, such as connectivity analysis or synaptic transmission simulations.

## 2 Methods

As stated above, this paper presents NeuroEditor, an application that offers a powerful framework for analysis and visual exploration of neuronal morphological data. It has been explicitly designed for the improvement of neuronal tracings, allowing users to edit, correct and compress their input tracings, independently of how they were generated or their specific features. This tool has been built upon other methods and tools previously developed in our group, including some that address the approximation of neuron membrane.

NeuroEditor’s visual environment simplifies the process of editing and correcting neuronal tracings. First, users are provided with representations of the original and modified tracings, being able to choose between different methods for visualizing the tracings and detecting and correcting errors. Also, the application offers a manual editing procedure, guiding users visually through the simultaneous representation of each neuron’s morphological tracing together with its reconstructed membrane, recovered and approximated from its tracing. Furthermore, since purely manual correction procedures tend to be tedious and time-consuming, different methods for the automatic detection and correction of errors and for the general improvement of tracings are also included in the tool. Last, users have the possibility of extending NeuroEditor with new features, by programming their own algorithms using Python and directly use them within the tool, without having to recompile the code.

### 2.1 Tool overview

NeuroEditor provides a visual environment for the exploration, analysis and editing of morphological tracings stored using the .swc format (Cannon et al., 1998). Once the cell data has been loaded, users can visualize the acquired tracing together with the cellular membrane, which is automatically computed, starting from the available tracing points. For that, NeuroEditor provides full 3D navigation capabilities, letting users both get general overviews of the cells under study as well as focusing and observing specific details, as desired. NeuroEditor also lets users to manually select and edit any of the tracing nodes, enabling them to introduce corrections in the tracing data. Additionally, a set of automatic methods for adjusting the sampling resolution is included in the tool.

The visual inspection of cell morphologies allows skilled users to detect the existence of errors introduced during the acquisition stage. However, this is a tedious and error prone task that can be assisted by the automatic error detection and correction procedures provided by NeuroEditor. For this purpose, the tool offers a set of tests that can be run for checking the occurrence of a variety of common errors, suggesting also possible correcting actions.

Finally, the tool can be easily extended by including user defined methods programmed in Python. This capability allows users to perform some operations, such as including new methods or modifying the parameters of currently implemented algorithms. The following sections give a detailed description of the tool functionalities.

### 2.2 Tracing data

NeuroEditor has been built upon NSOL, a library that provides data structures for handling basic neuroscientific data, such as neuron morphologies (Vg-Lab, 2015). Internally, the morphology includes the descriptions of the soma and the set of neurites that integrate each neuron: dendrites (such as basal and apical dendrites, if pertinent), and axon. Each neurite is described by a set of points extracted along its trajectory (referred to as *nodes*), where each point stores its spatial coordinates and the neurite radius on that position. Special attention is devoted to bifurcation points since they determine the branching patterns of neurites. Also, the branches or sections are composed of *segments*, where a set of segments or a section is found between two bifurcation points. All this data can be recovered by NSOL from morphological tracings stored in .swc file format (Cannon et al., 1998).

As output, NeuroEditor can save both the modified morphological tracings and the 3D meshes approximating neuronal membranes; the tracings are stored also as .swc files, and the 3D meshes, as .obj files. These meshes are generated with NeuroLOTs, a library (Vg-Lab, 2020) that allows the generation and visualization of meshes approximating cell membranes using the approach presented in Garcia-Cantero et al. (2017).

### 2.3 Visualization

As commented above, NeuroEditor provides an interactive 3D environment for the inspection, editing, and repair of morphological tracings. For facilitating this task, NeuroEditor provides feedback to users regarding the results of the tracing modification process, allowing the comparison of the original and edited tracings. Being able to count with the interactive membrane mesh generation capabilities that NeuroLOTs provides, any modification carried out on the tracings can be immediately incorporated into the membrane mesh generated by NeuroLOTs. The newly computed membrane can then be presented to the user, together with the original and modified tracings. Both tracings are displayed as sets of polylines, where each point of the tracing is represented by a sphere, whose radius coincides with the thickness of the neurite at that point, according to the tracing.

**Figure 1:**
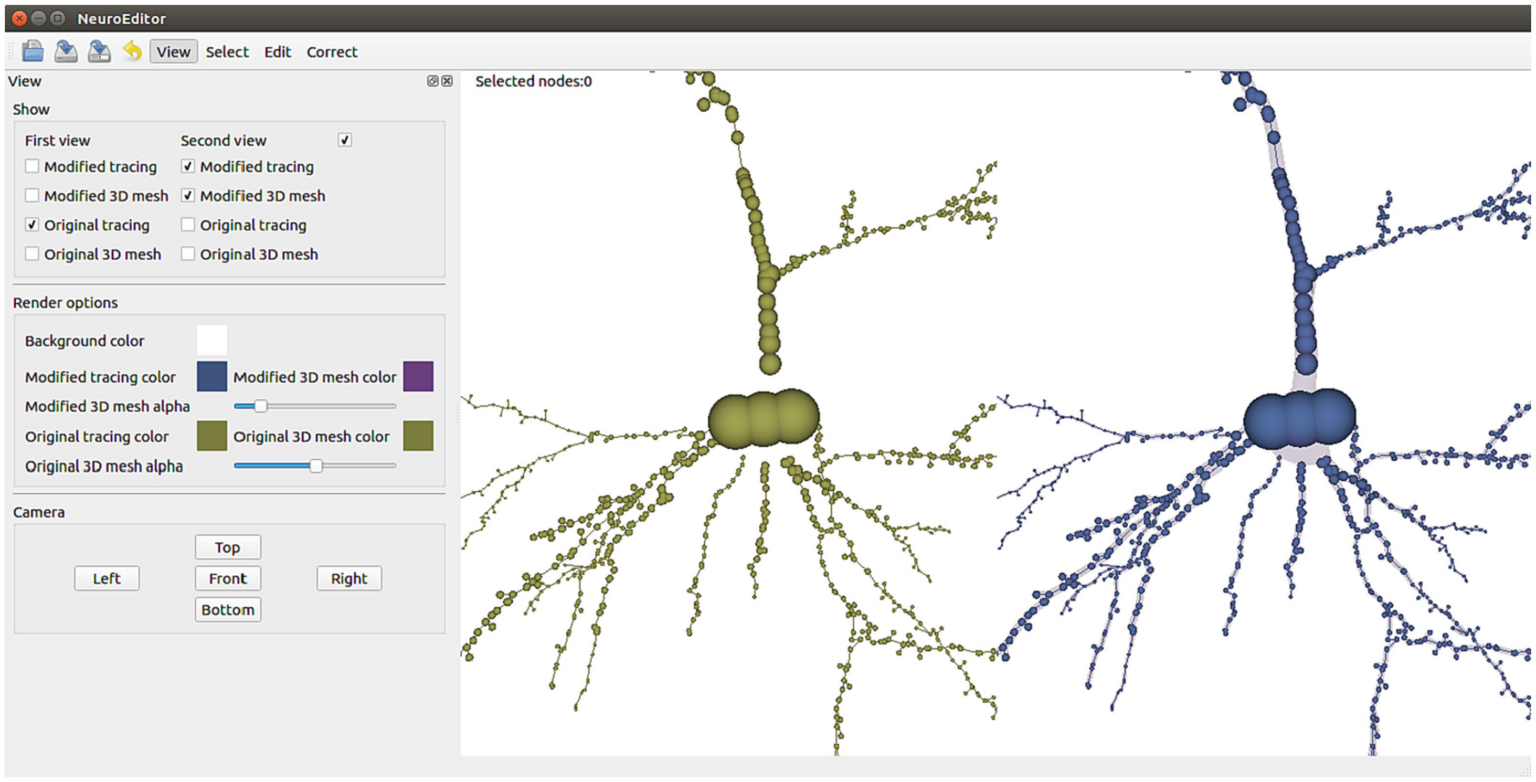
NeuroEditor 3D visualization properties panel and visualization window.

To facilitate the tracing editing process, the original and edited tracings can be visualized side by side or even superimposed for comparison purposes. The polygonal mesh that approximates the cell membrane can also be laid over the tracing, applying transparency to allow viewing both structures simultaneously. Figure 1 shows a 3D visualization example, having the original tracing on the left and the modified tracing with the 3D mesh approximating the membrane on the right. The left panel in the figure presents all the configuration options that the user can tune to select which elements to visualize and to parameterize certain visual properties such as color and transparency.

Since the generation of the membrane mesh follows the approach presented in Garcia-Cantero et al. (2017), a synthetic yet plausible 3D soma shape can be visualized, even if the tracing does not provide sufficient information to recover its original anatomy. This 3D approximation can be computed following the deformation process described in Brito et al. (2013) and Garcia-Cantero et al. (2017) and is used only for the generation of the membrane mesh for its visualization. It must be pointed out that any computations performed for visualization purposes will not modify the tracing data, but any changes introduced by the tracing editing procedures will indeed modify the visualization presented to the users in real time, as well as introduce changes in the tracing data that can be saved in the edited version of the cell tracing.

### 2.4 Selection tools

**Figure 2:**
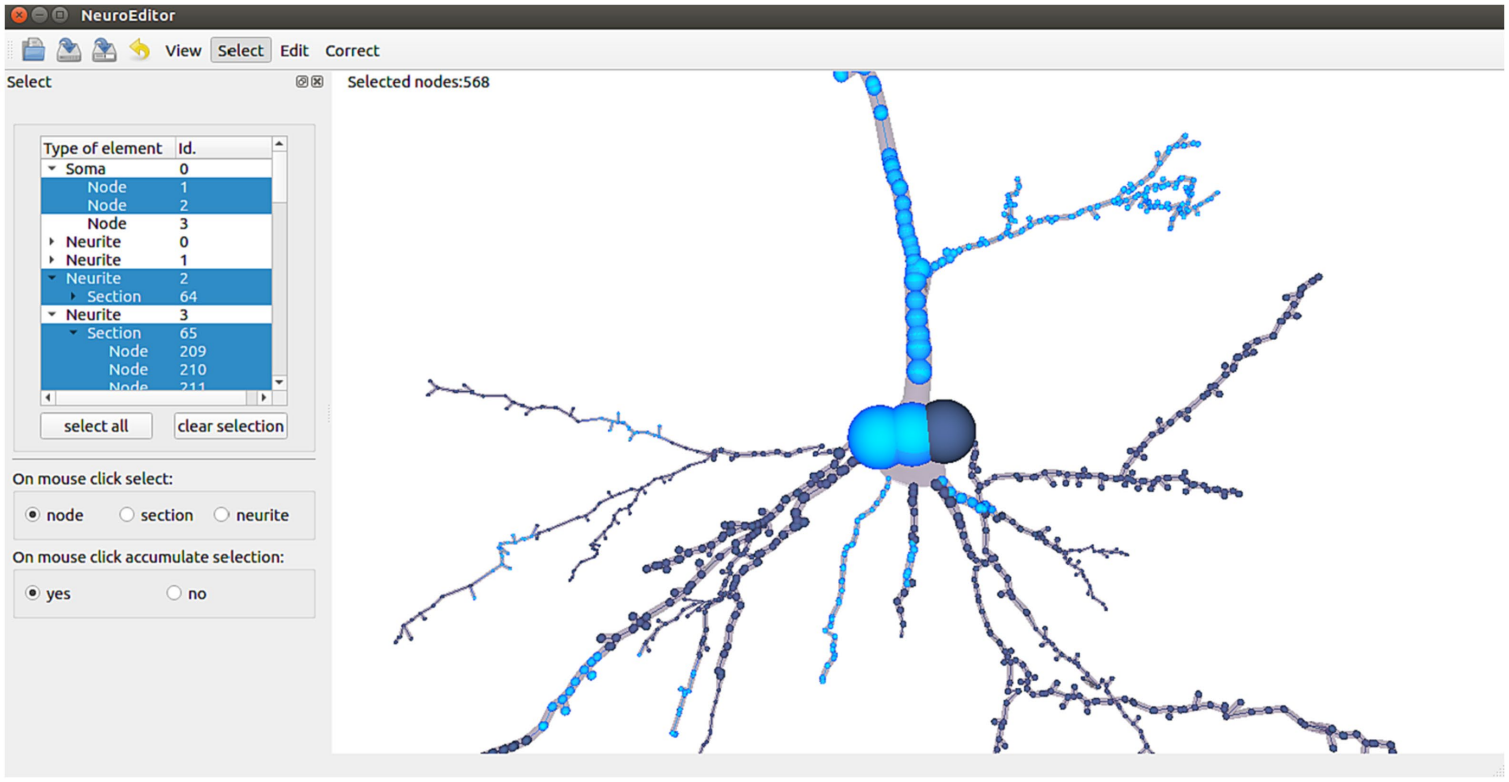
NeuroEditor user interface for the hierarchical selection or 3D mouse selection of elements. Highlighted nodes in the tracing (in light blue color) correspond to the selected elements in the left panel (also in blue color).

NeuroEditor allows users to interactively select elements from each tracing in two different ways: navigating through the hierarchy of morphological elements (left panel in Figure 2) or selecting the desired ones with the help of the mouse while navigating through the views depicting the morphological tracing (Figure 2, right panel). In the first case, the hierarchy of elements is represented in a tree viewer that initially shows the soma and the first order neurites of the cell under consideration. Each of these elements can be recursively expanded to show its sections and its tracing points (left panel, Figure 2).

This hierarchical structure allows performing fast selection operations at different scales, since clicking on a high-level entity automatically selects all of its descendant entities. This way, selecting a specific neurite implies selecting all of its sections and tracing points.

If the selection is performed with the mouse over the 3D tracing, multiscale selection is achieved by letting the user specify the mouse-clicking behavior in terms of selection scale. This means that the user can specify if clicking on a node may imply selecting only the node, the whole section that includes that node or the whole neurite the node belongs to (left panel in Figure 2).

In order to simplify the selection of multiple elements, selections can be configured to be accumulative; this means that the result of a selection is the combination of the newly selected elements plus any other elements that had been previously selected.

### 2.5 Editing operations

The editing capabilities provided by NeuroEditor allow the modification of different tracing features, which in turn results in the improvement of the tracings quality. Several editing strategies have been implemented, such as the manual editing of the tracing nodes properties, the automatic simplification or refinement of the tracings, the automatic detection and correction of a predefined catalogue of errors, and last, the possibility of executing user written python code to modify the tracings. The following sections explain these approaches in detail.

#### 2.5.1 Manual editing of selected elements

**Figure 3:**
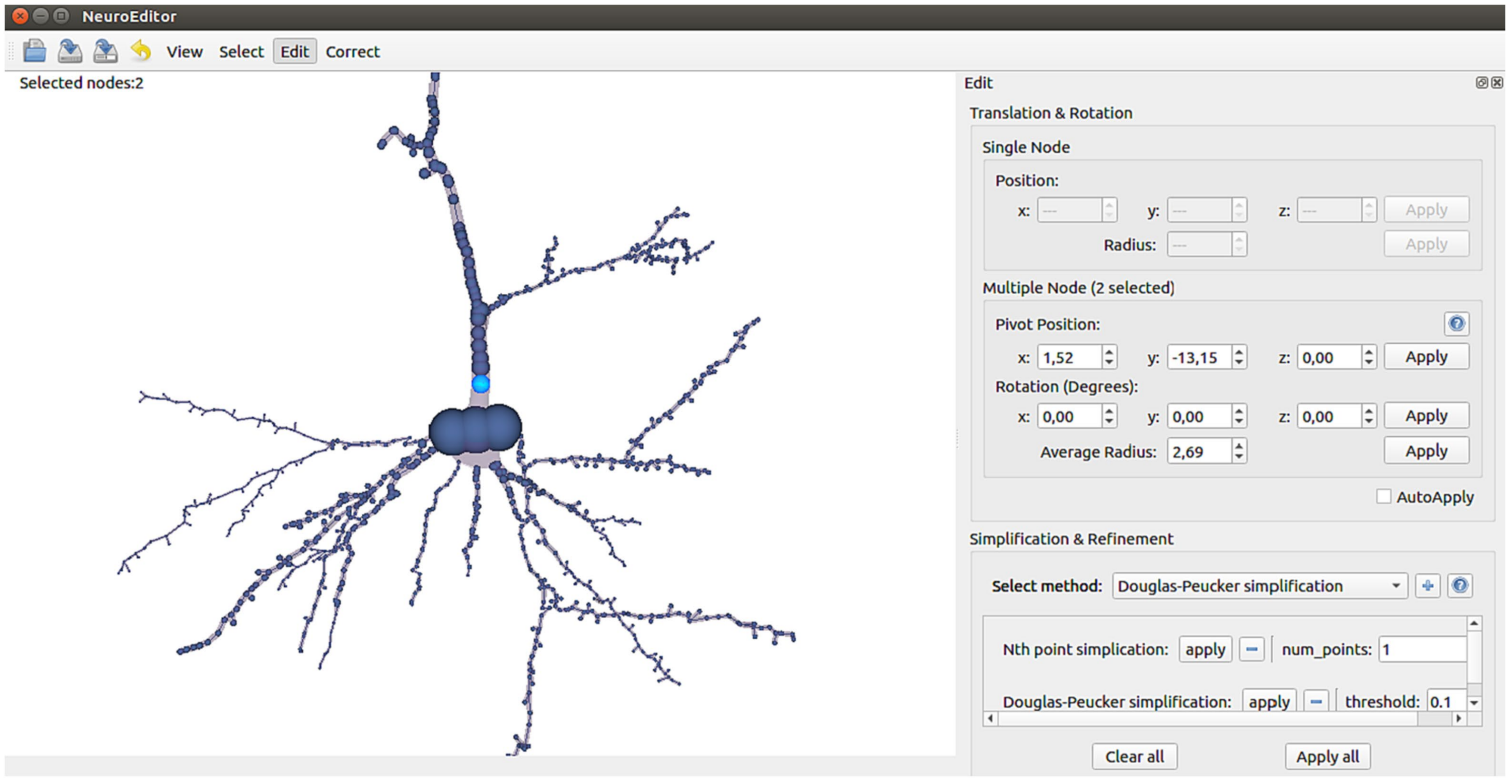
NeuroEditor manual editing, simplification and refinement interface panel.

Users can freely modify the position, rotation (disabled for single node selections) and radius of any tracing node or group of nodes. First, the nodes to be modified must be selected using any of the selection tools previously described. Then, the editing panel (top of right panel in Figure 3) will show the current position, rotation angle and radius of the selected nodes, allowing users to type new values for updating them.

In case of multiple selection (more than one single node selected), then the editing panel will show the average values for each of the properties. Editing an average value results in updating the single values of all the selected nodes (proportionally) in order to obtain the new average value.

#### 2.5.2 Simplification and refinement operations

NeuroEditor provides several simplification and refinement methods that can be applied to neuron morphological tracings. Each method has its own parameters, and they are user-configurable (bottom part of the right panel in Figure 3). If the user has selected a set of nodes, the processing operations specified by the user will be carried over the morphological sections whose nodes are completely or partially included in that set. If no nodes were selected, the methods will be run over the whole tracing.

**Figure 4:**
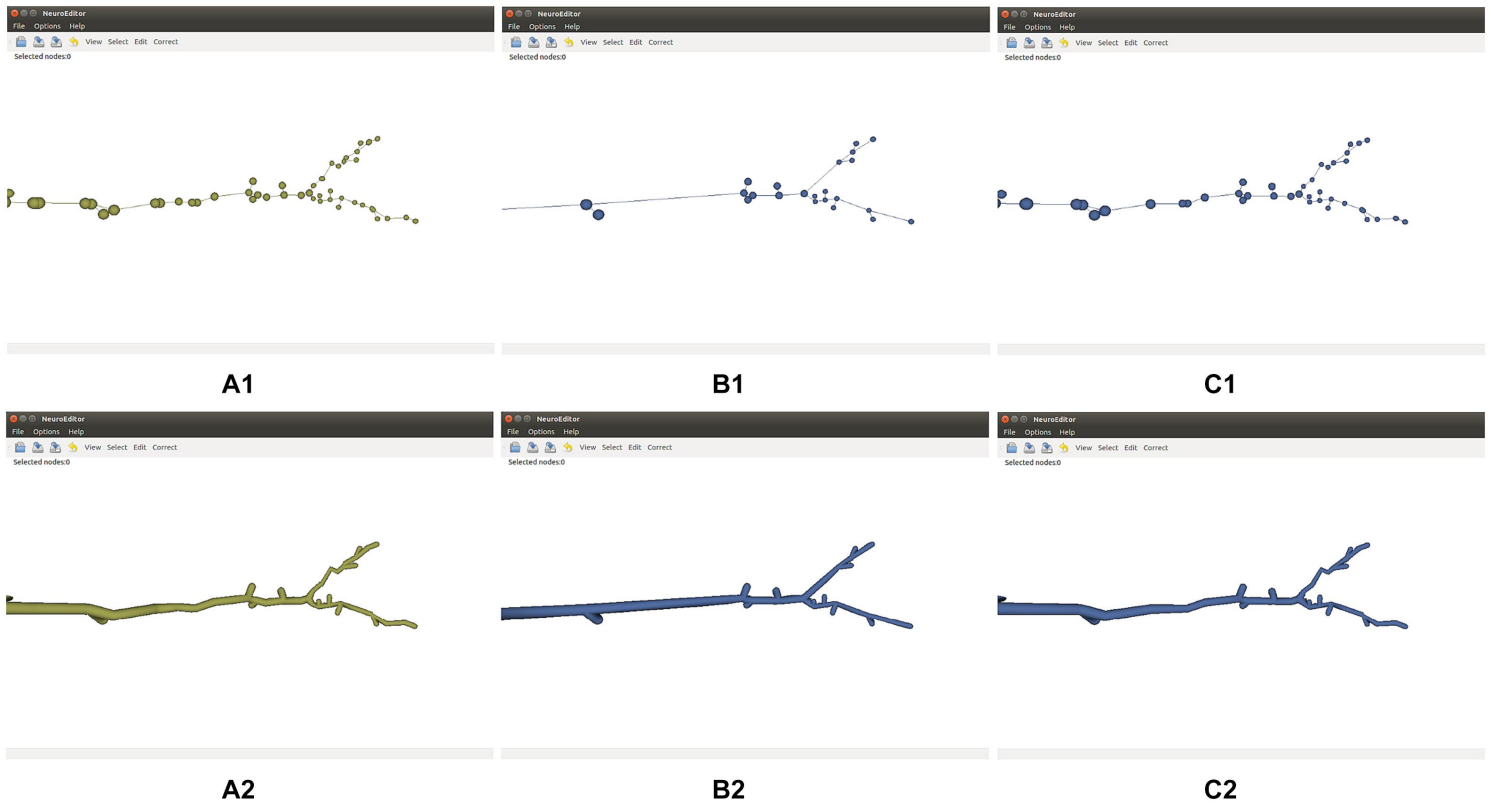
Effect of two different simplification methods. Top row: initial tracing **(A1)**, its simplification after applying Douglas-Peucker’s algorithm **(B1)**, and its simplification after applying the Perpendicular Distance algorithm **(C1)**. Bottom row: 3D mesh approximating the membrane of the initial tracing **(A2)**, and 3D membranes of the simplified tracings displayed **(B2,C2)**.

Tracing simplification is advisable whenever the tracing trajectories have been oversampled. Since the morphological sections can be considered as equivalent to polylines, the simplification algorithms used here follow strategies common for polyline simplification (Shi and Cheung, 2006). Specifically, the following simplification techniques are distributed with NeuroEditor: Nth point, Radial distance, Perpendicular distance, Reumann-Witkam; Ophein, Lang, and Douglas-Peucker (psimpl, 2024). Figure 4 illustrates the effect of applying the Douglas-Peucker N or Perpendicular Distance methods to an initially oversampled neurite section.

Refinement operations permit adding new morphological points to a tracing for increasing its sampling density, something that may be useful for adapting the tracing resolution to the needs of compartment-based simulations. Given the tracing point density (number of tracing points per unit of length), two refinement options are already implemented in NeuroEditor:

- Linear interpolation sampling. This option inserts new tracing points in each segment until the tracing point density reaches the value specified by the user. The neurite trajectories will not be changed, although they will be defined using more morphological points.
- Cubic interpolation sampling. This approach inserts the new tracing points following a Hermite spline, smoothing therefore the neurites’ trajectory.

**Figure 5:**
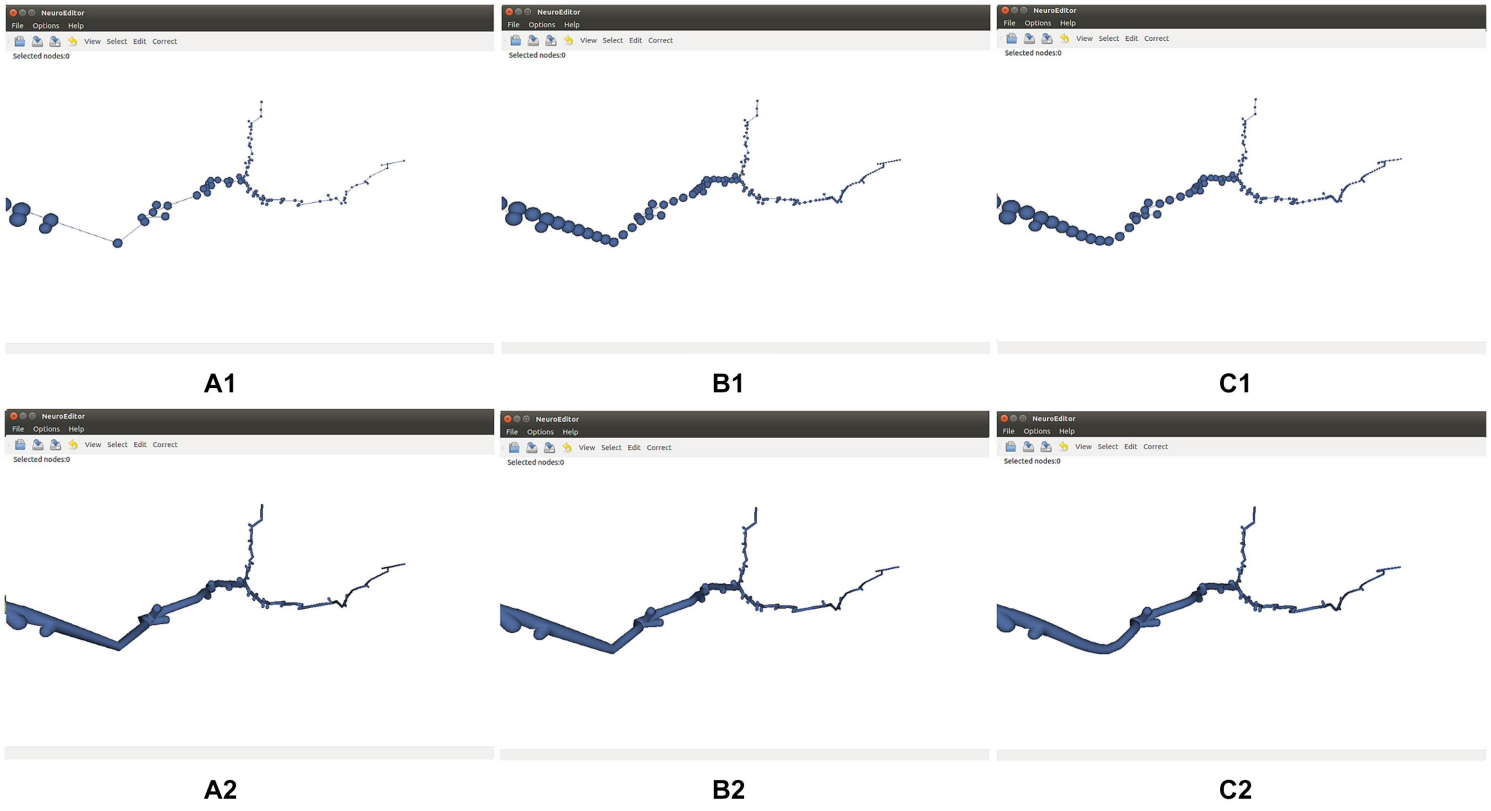
Effect of refinement methods. **(A1,A2)** Initial tracing and its corresponding 3D mesh approximating the cell membrane. **(B1,B2)** Refined tracing and 3D mesh computed applying linear interpolation. **(C1,C2)** Refined tracing and 3D mesh computed applying cubic interpolation. Both refinements are applied with a parameter of 0.5 points per unit of length.

Figure 5 shows the effect of these two refinement approaches on a neurite section. Cubic interpolation (Figure 5C) renders smoother trajectories that are visually more pleasant.

#### 2.5.3 Automatic detection and correction

Morphological tracings can be visually inspected for evaluating the overall quality of the extracted skeletons and for detecting different eye-catching undesirable artifacts. However, the process of visual inspection is tedious and error prone. Automatic error detection procedures improve error detection rates and user workload, also facilitating the automatic application of sets of correction actions.

**Figure 6:**
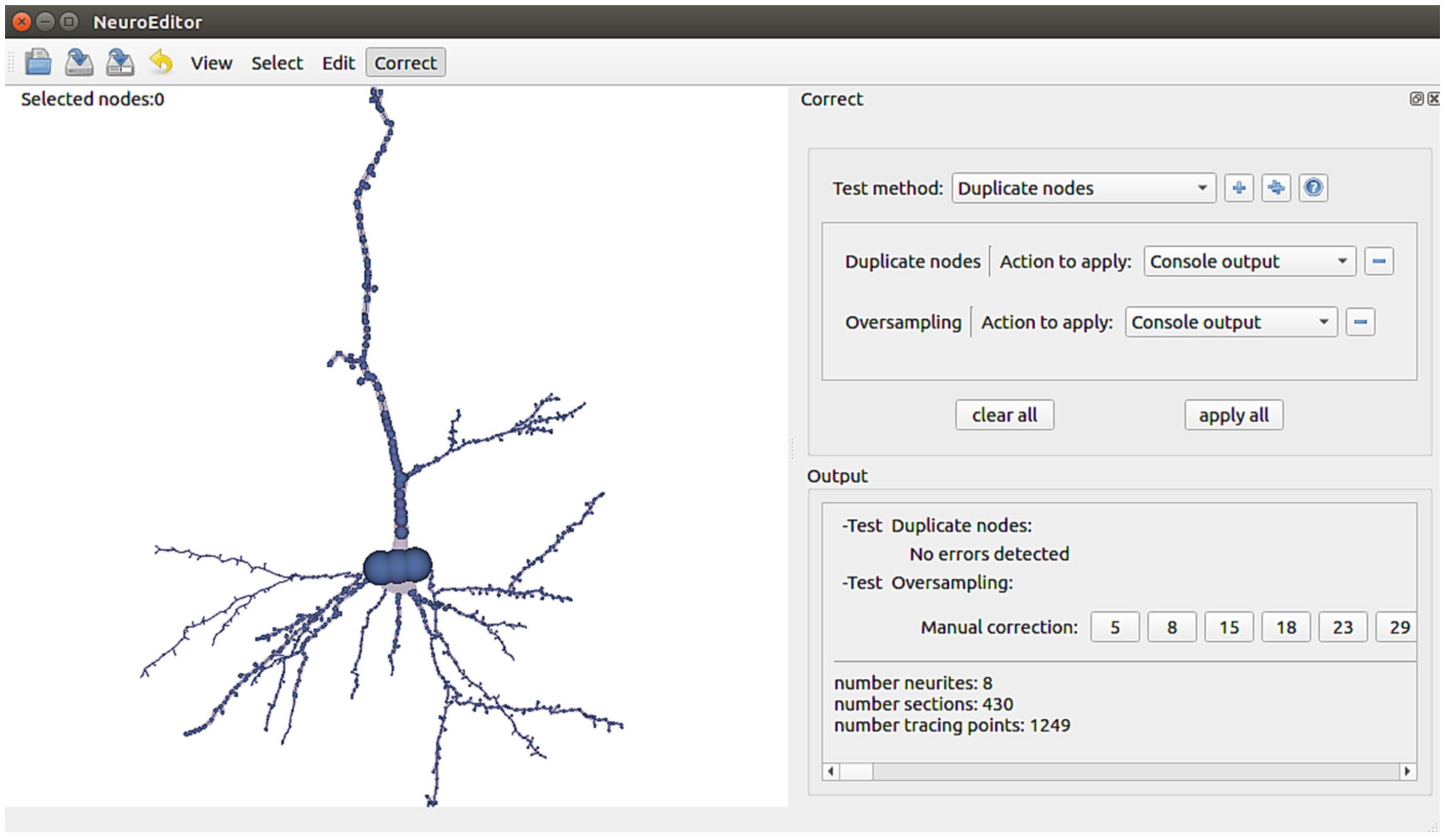
NeuroEditor interface panel for automatic tracing error detection and correction, departing from a catalog of errors identified as frequent.

NeuroEditor provides a set of algorithms that allow automatic identification of some kinds of potential errors. In addition, the tool implements a set of actions that the user can carry out to correct them (Figure 6). It must be noted that not all the potential errors are suitable for automatic correction, since some of them require user supervision, either for the evaluation of the situation or for deciding the most appropriate solution. The following paragraphs present a catalog of the errors that are automatically detected nowadays and their possible solutions, which are already implemented in NeuroEditor. In addition to these algorithms, users can implement their own methods, as it will be explained in the section “User-defined operations.”

**Figure 7:**
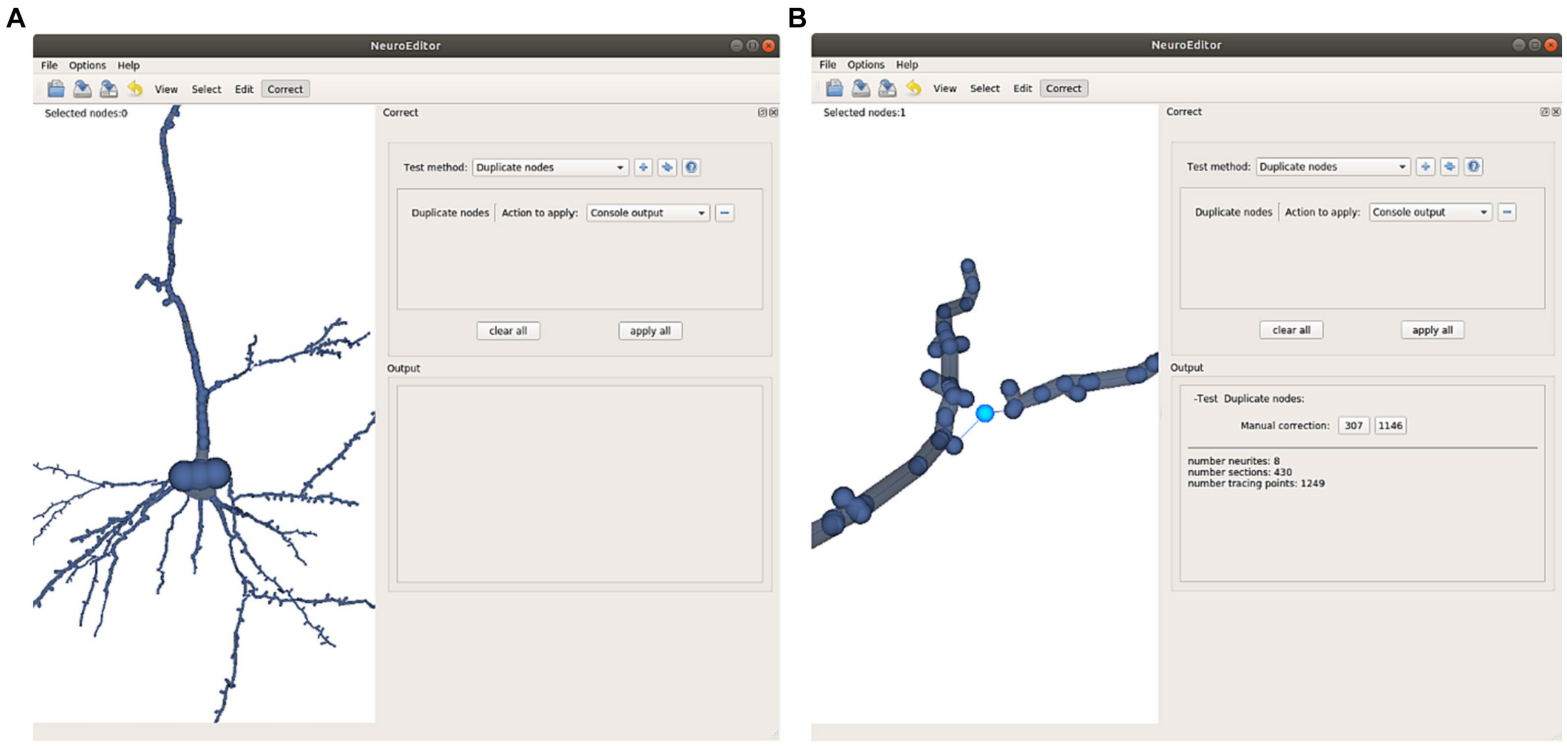
This Figure illustrates the automatic detection and manual correction process of duplicated nodes. **(A)** The user selects the “duplicate nodes” test method and the “console output” action. **(B)** A notification of the detected repetition is notified in the application console; clicking on the notified node’s number selects the morphological point on the image and focuses the camera to obtain a close view. Now the user can decide either to remove the repeated node or to displace it to a different position.

**Figure 8:**
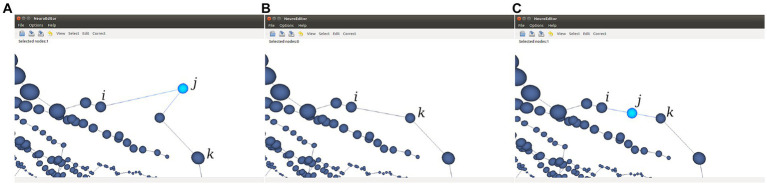
This figure illustrates the two options provided for the automatic correction of sharp changes in neurite trajectories. **(A)** Initial situation where the segment j-k generates an angle below 90° with the previous segment j-i. **(B)** Node j is removed and a new segment is created from node i to node k. **(C)** Node j is displaced to the middle point of the virtual segment that connects nodes i and k.

- Repeated nodes. This test checks whether two tracing nodes are overlapping, sharing the same 3D position. Whenever this situation occurs, one of these three correction actions can be taken: Notifying the error in the console output, removing the duplicate nodes, or repositioning one of the duplicated nodes into a new position, computed as the middle point of the incident segment (Figure 7).
- Oversampling. This method analyzes if the distance between two consecutive tracing points is below a certain threshold, a situation that may suggest that the neurite trajectory is oversampled. This threshold can be tuned using the customization capabilities provided by NeuroEditor. Presently, the oversampling threshold in the test is fixed to be equal to the sum of the segment nodes radii (this can be used as an automatic criterium for detecting oversampling, although other criteria can be introduced). The options presented to the operator for processing these errors are the same as those provided for correcting duplicate nodes: console output, removing nodes below the specified threshold or moving the node to the middle point of the segment under consideration.
- Tracing points inside the soma volume. This test checks whether any of the tracing points that describe the neurite trajectory falls inside the soma volume. The soma volume is approximated by a sphere whose radius and center are computed from the soma description; tracing points inside this volume may lead to artifacts in the deformation process that the sphere will undergo while the cell membrane is being generated. This undesirable situation can be induced by an inaccurate acquisition of the tracing or by the inexact initial approximation of the soma shape. In consequence, the possible actions provided for correcting this error are console output to let the user perform any manual adjustment, such as editing the soma description or the nodes’ properties; removing the nodes inside the soma volume or moving the nodes that are inside the volume to the soma surface.
- Neurite initial segments too distant from the soma. NeuroEditor evaluates if the first tracing point of each neurite is within a reasonable distance from the soma by setting a distance threshold relative to the soma radius. Presently, this threshold has been fixed to a value of 4 times the soma radius. As before, this value can be changed by the user creating a customized method with a different threshold. The available actions to deal with this situation are console output to let the user perform any manual adjustment or editing the soma description and repositioning the first node of each neurite on the soma surface.
- Sharp changes in neurite trajectories. This test checks if two consecutive segments represent an abrupt change in the direction of the neurite trajectory (Figure 8A). In case the angle between the two segments is below certain threshold (90° in the implemented method), then three possible correcting actions are provided: console output; deleting the “outlier” node (keeping the first node of the first segment and joining it with the final node of the segment that forms a sharp angle with it; Figure 8B); or displace the “outlier node” to the middle point of the new segment that replaces the two original ones (Figure 8C).
- Parent neurite’s radius smaller than child neurite’s radius. This test checks bifurcations to assess that the radius of each children segments is not larger than the radius of their parent neurite. If the parent’s neurite radius is smaller than any of the children’s radii, a warning is emitted, so that the user can decide the action to be taken: editing the radii and/or coordinates of the nodes, or approving it as being correct, for example.
- Constant radii along the neurite. In case the radius does not vary for all the tracing points of a neurite, a console output is generated for users to decide the action to be taken.
- Interpenetration in bifurcations. This test checks all the bifurcations in order to evaluate if the radii of the children’s neurites are coherent and do not generate collision between their corresponding membrane segments, something which results in membrane interpenetration problems. If this situation occurs, a notification is also generated in the application console.

In terms of performance, the total time taken by the tool to perform all automatic error detection tasks has been measured for 5 neurons obtained from EBRAINS (2024) with different number of tracing points. The results are shown in Table 1. As can be seen, the tool performs these tasks in real time, taking only 63 ms to process a neuron with 19,300 tracing points. In addition, we have also measured the performance of the automatic error correction, where for the neuron with the highest number of tracing points (19,300) required only 10 ms.

**Table 1:** Execution times of all automatic error detection algorithms for 5 different size neurons.

Neuron	Tracing points	Execution time (ms)
53 Human hcca1 idab1 porta4 sec1 cel18	2,576	8
10 Human hcca1 idab1 porta5 sec1 cel7	5,629	20
11 human hcca1 idab 1 porta5 sec1 cel8	7,928	18
7 human hcca1 idab1 porta4 sec1 cel11	19,300	63

#### 2.5.4 User-defined operations

**Figure 9:**
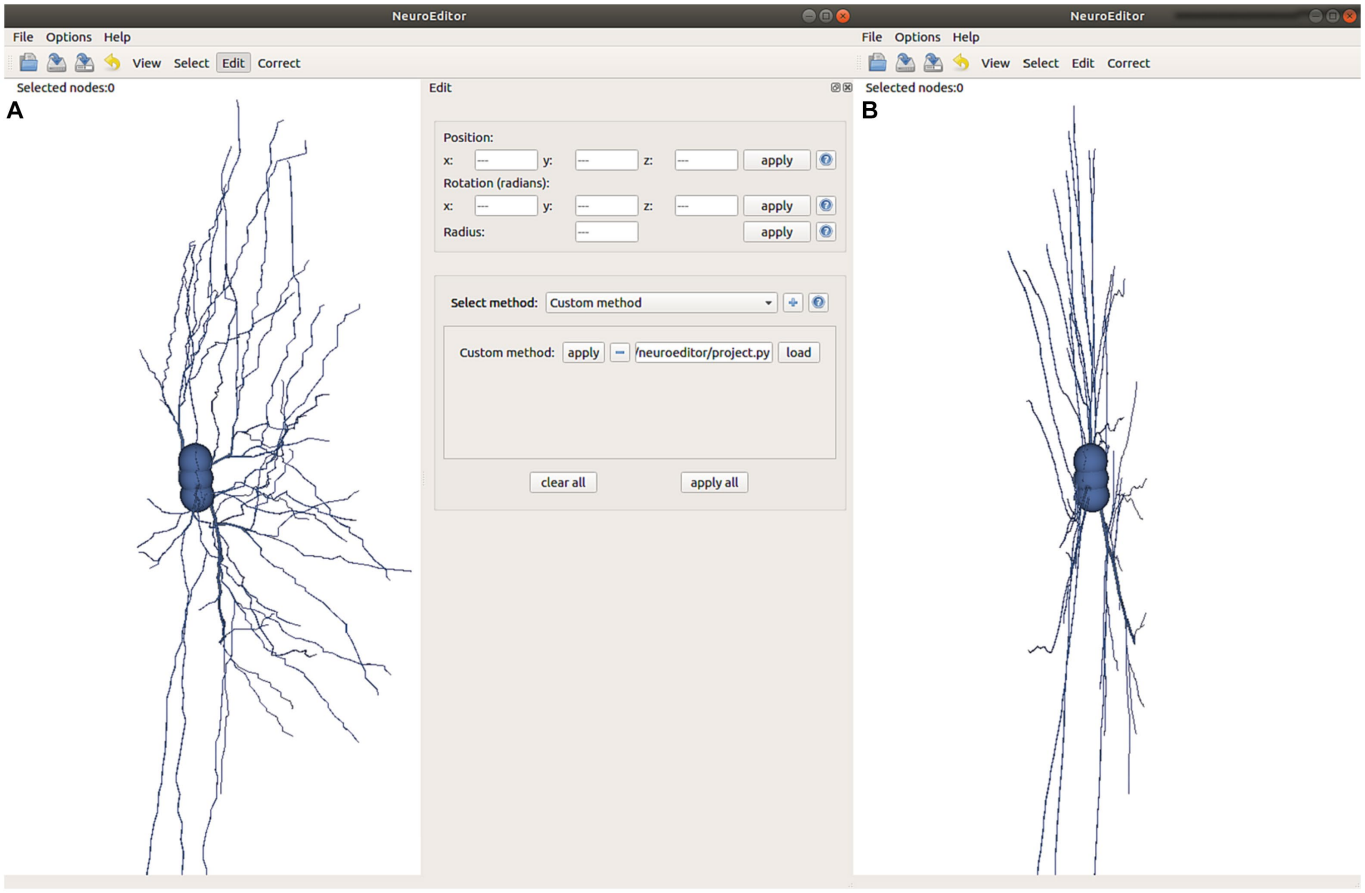
This figure illustrates the effect of executing the custom method defined in Figure 10. **(A)** Visualization of the original tracing and selection of the user-written program to be applied. **(B)** Modified tracing where all the tracing points have been projected onto the XY plane with coordinate z = 0.

In addition to the set of algorithms already implemented and distributed in NeuroEditor, users can extend the tool functionality by programming their own algorithms in the Python programming language. This way, user-programmed algorithms can be iterated over all the neuron sections, carrying out the user-defined functionality. For this purpose, users just need to select the option “custom” in the right panel in Figure 9, specify the file containing the code to be executed and apply it similarly to any of the other methods described in the previous section. There is no need to recompile the code or to know the application architecture.

**Figure 10:**
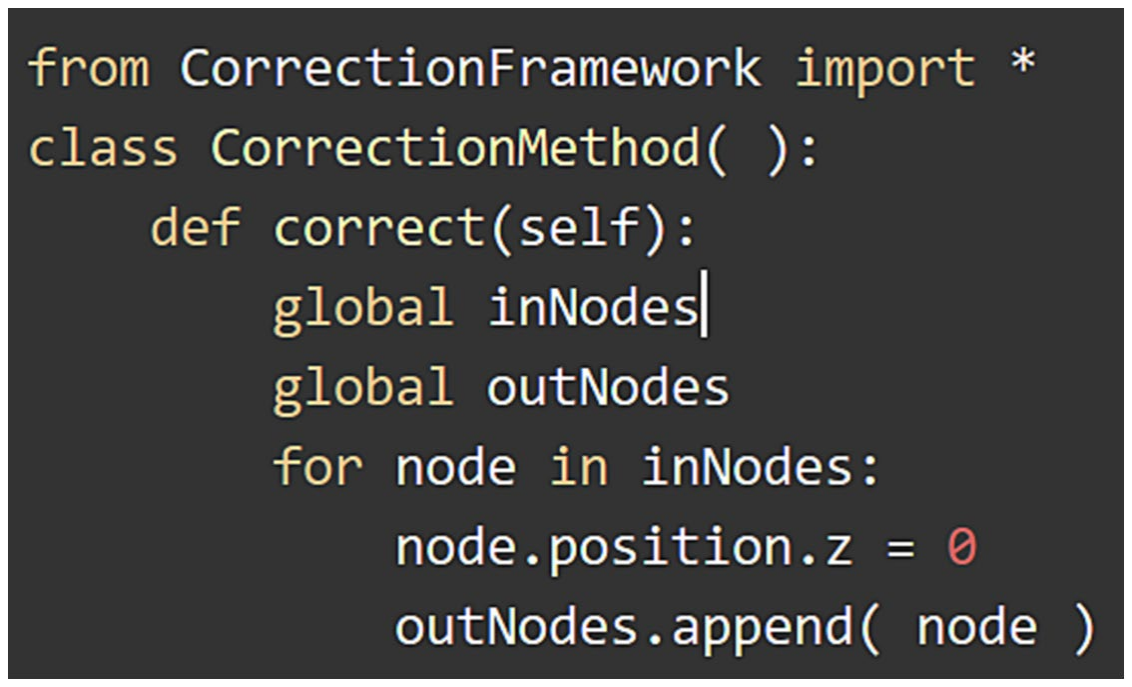
Example of custom method that sets coordinate *z* to 0 for each tracing point.

In order to develop a custom method, users need to specify the tracing area section (the set of nodes) where the custom method will be applied, as well as the algorithm to be run. The input section is defined as a list of nodes (inNodes), and the outcome of any custom method will be the list of modified nodes for the section that is being processed (outNodes). The custom method will be able to access and modify any of the nodes’ properties (position, radius and identifier). Figure 10 shows an example of a user-written method, where all tracing points are projected in the X-Y plane along the Z direction. It must be noted that by creating the output list of nodes, the custom method can also modify the sequence of nodes that define the section.

This example replicates every node in the input list and modifies its z coordinate by setting it to 0, projecting therefore the nodes onto the X-Y plane. After that, the modified node is appended to the output list of nodes. The effect of running this custom method can be observed in Figure 9B.

## 3 Processing neuronal tracings with NeuroEditor

This section illustrates the combined application of some of the tool features and capabilities by means of an example, in which real neuronal data is used. In fact, the data used in this example come from the morphological tracing repository NeuroMorpho (Halavi et al., 2012). Many neuron morphological tracings, included in this and other repositories, present several tracing errors derived from the different acquisition techniques and procedures applied to acquire them.

The following subsections show a hypothetical workflow, describing a sequence of steps for loading, inspecting, editing, and correcting a morphological tracing. Obviously, this is just an example involving decisions that should be taken by the real user depending on his final goal.

The tracing used in this example belongs to a pyramidal neuron from the anterior cingulate of the neocortex corresponding to an adult human (02b_pyramidal1aACC–NeuroMorpho) that presents some typical features such as an incomplete description of the soma and oversampling in certain dendritic sections. This morphology will be processed, combining manual editing of the soma description, automatic detection of super-sampled sections, automatic adjustment of the resolution and the execution of a user defined method for setting a uniform thickness along the dendrites.

This processing pipeline exemplifies a possible sequence of operations to correct and homogenize morphological descriptions that can be subsequently used for further analysis or comparative studies.

### 3.1 Manual editing

Loading the morphological tracing into NeuroEditor is the first step of the pipeline. Once the desired cell morphology is loaded into the application, the user can manually modify any of the points that comprise the neuron morphological tracing. This manual editing operation can be visually guided by the neuron membrane mesh representation, since this mesh is reconstructed immediately, reflecting any modification applied to the neuron tracing.

**Figure 11:**
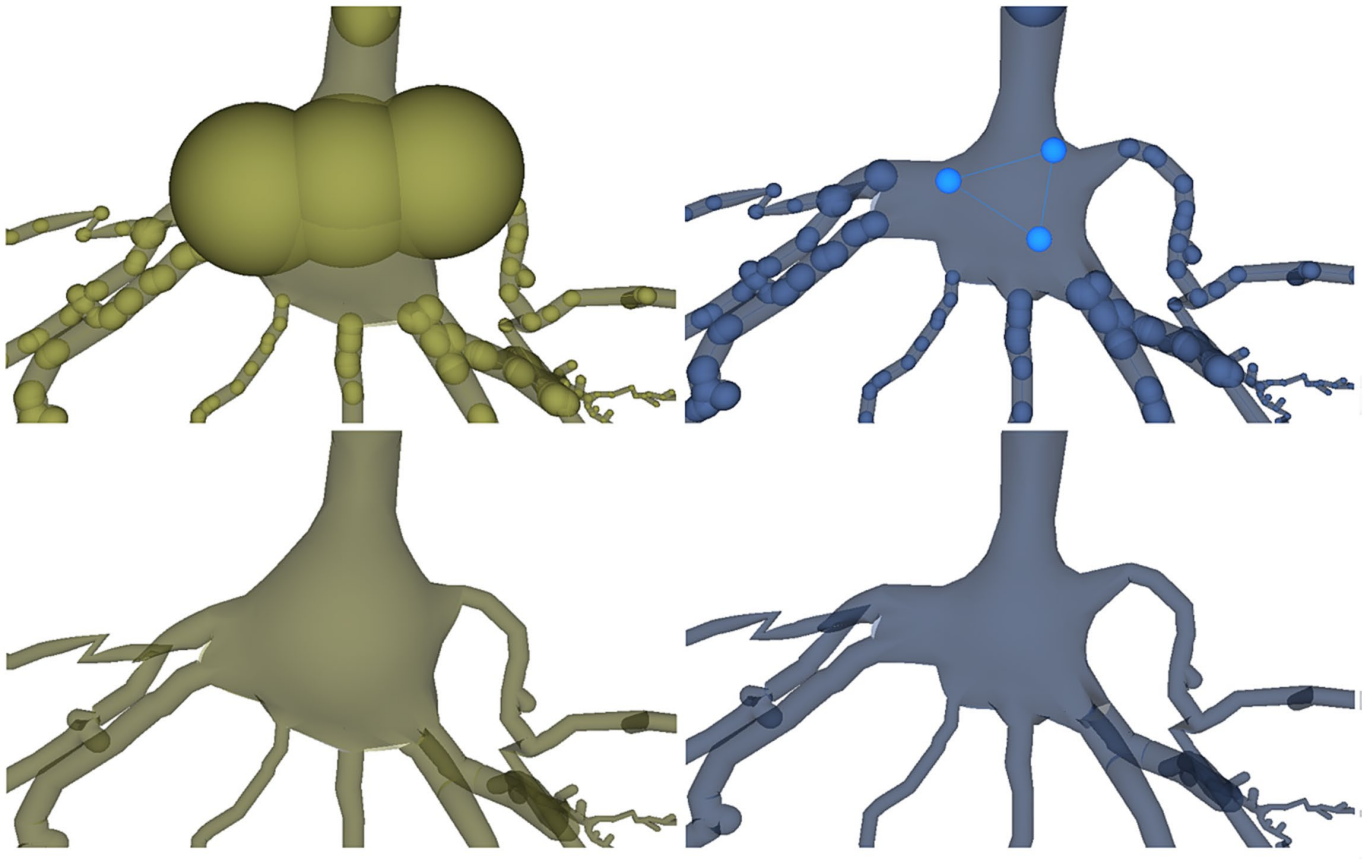
Modification of the soma description within the tracing. The left images show the original soma tracing in yellow with three tracing points and its reconstructed mesh. The right images show in light blue the modified soma tracing, described now with the three points arranged in a triangle to obtain a more accurate reconstructed mesh based on the neurites’ beginnings.

After visually inspecting the morphology, the user could decide to edit any of the elements describing the neuron. Let us assume that, in this case, the shape of the soma is not satisfactory, and the user decides to modify the description of the soma by editing its tracing points. Figure 11 shows the result of the user modifications regarding the tracing points that describe the neuron soma as well as the process followed for introducing these modifications. First, the user is presented with three aligned points with large radii describing the soma (left) and decides to manually adjust their radii and their positions in order to adjust the soma reconstructed mesh shape taking in to account the neurites beginning positions. As mentioned before, during this process the user can be seeing the mesh generated for the soma while being interactively modified as he modifies the tracing description to obtain the desired shape.

### 3.2 Automatic detection and correction of tracing errors

After introducing manual modifications such as the soma modification described above (in case the user decides to perform any), he can continue processing the morphological tracing under consideration by running any of the automatic procedures provided by NeuroEditor, which have been specifically developed for correcting frequent errors appearing in tracings. If the user decides to carry out any of these procedures, then he can subsequently select the most appropriate action for correcting each detected error.

**Figure 12:**
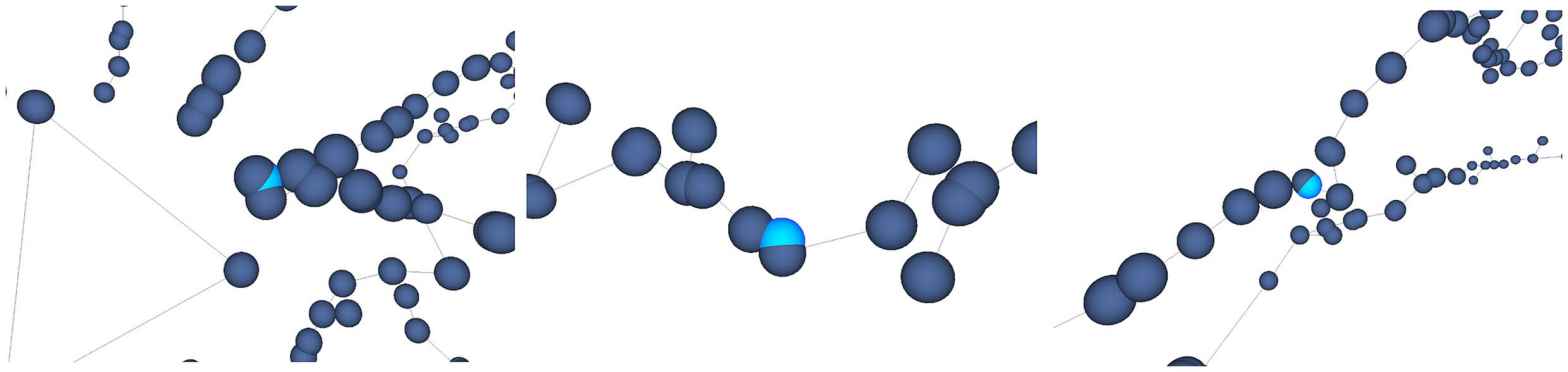
Automatic oversampling error detection. Oversampled nodes are located and highlighted in light blue, as shown in this figure. Different options are offered to the user, who can decide then the most appropriate action to be taken with respect to these nodes.

For example, Figure 12 shows some results achieved after running the Oversampling test on the selected neuron. In this case, the user is presented with the nodes labeled as erroneous, highlighted in light blue. The user is then able to decide which action will be taken on those nodes, such as applying some automatic correction procedures or deleting the considered nodes.

### 3.3 Resolution adjustment

The next step taken in this example is the re-sampling of the tracing points. This process can use any of the different simplification or interpolation methods implemented in NeuroEditor. Remember that the goal of these methods is to allow users to increase or decrease the number of samples that define the tracing sections, in order to adequate the tracing resolution to their specific needs.

**Figure 13:**
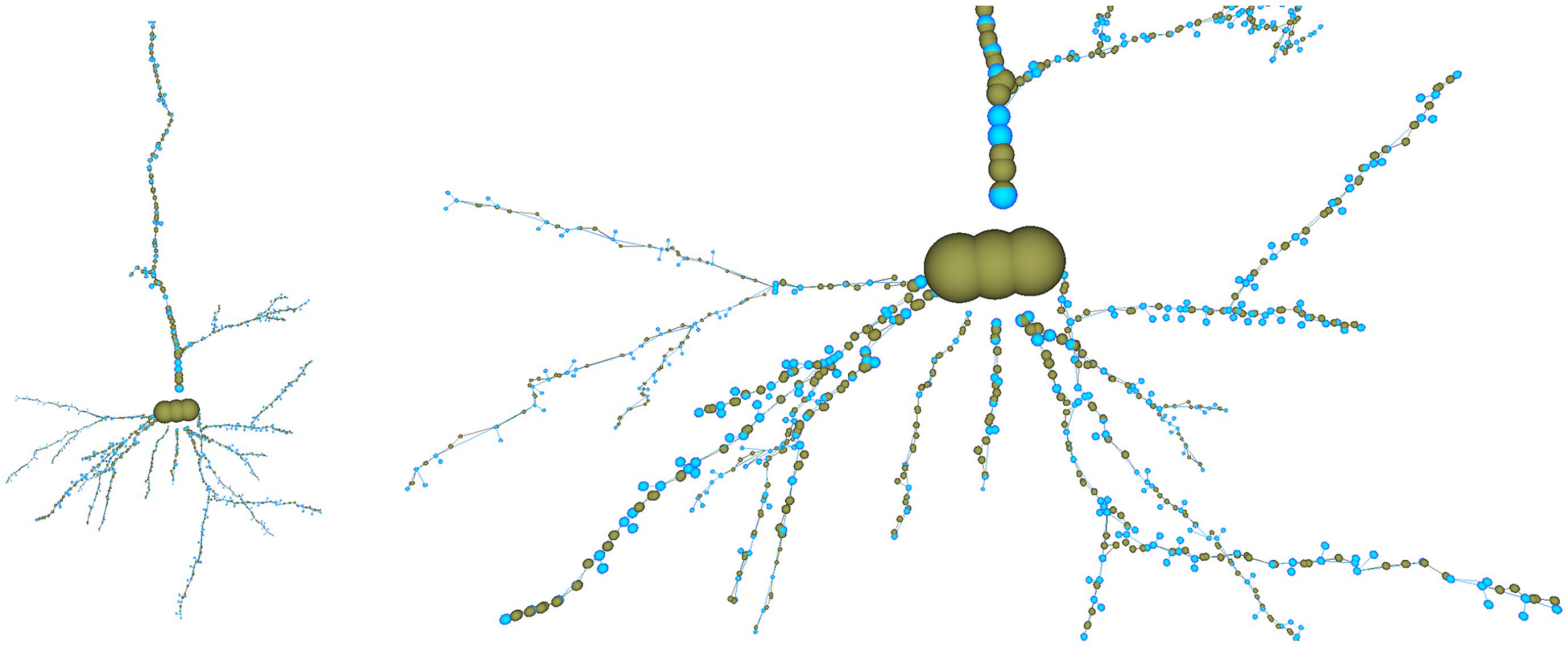
Overall view of the cell tracing (left) and close view of the basal dendritic tree (right) after reducing the tracing resolution by applying a “Nth simplification” procedure with a simplification factor of 4. The nodes discarded from the original tracing are shown in greenish yellow, and the nodes kept in the modified tracing, in light blue.

For example, if the user wants to reduce the number of tracing points that compose the morphological tracing, he can choose among different options. In this case, the user has decided to automatically reduce the number of tracing points by applying a “Nth simplification” procedure, with *n* set to a value of 4. We refer to the points on the tracing between two consecutive bifurcations as a section. This simplification method discards three out of each four tracing points, except for the first and last points in each section that are never discarded. As a consequence of this process, only 562 of the original 1,249 tracing points are kept, resulting in an appreciable simplification (please note that simplification takes part per section, keeping always the first and last nodes in the section). Nevertheless, the neuron most relevant morphological features are kept, as can be seen in Figure 13, where the discarded nodes are represented in greenish yellow.

### 3.4 User defined correction method

The last step in the correction pipeline presented in this example is the application of user-developed custom correction methods over the tracing under consideration (as explained before, users can introduce processing methods coded in Python). As in the previously presented pipeline steps, the results of this custom-coded correction are interactively reflected over the reconstructed mesh, giving users immediate feedback for the visual validation of the applied corrections.

**Figure 14:**
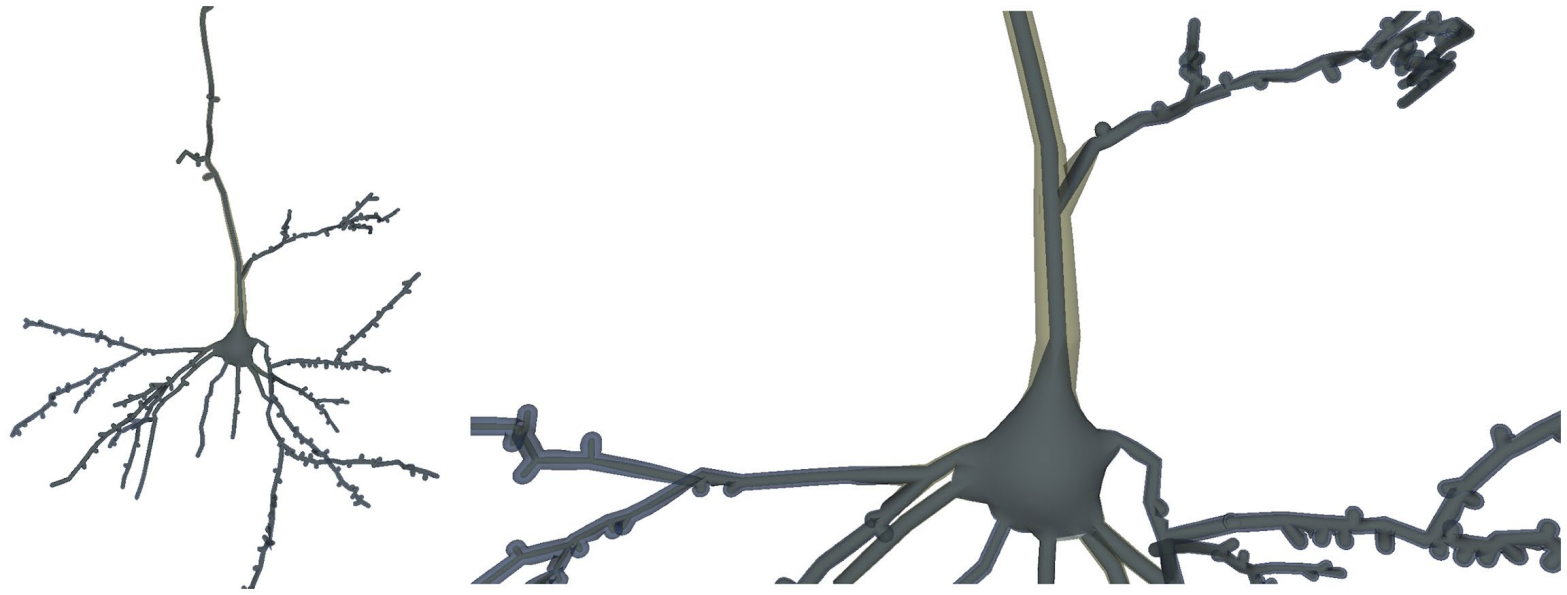
Neuron morphological tracing modified by the custom-developed method as described in this section, setting the thicknesses of all the tracing points to 1.0. The mesh reconstructed from the modified tracing is depicted inside the mesh reconstructed from the original tracing, for comparison purposes. On the left, the whole neuron is displayed, while the image on the right is a close-up view of the same neuron.

In this example case, the user wants to modify the radii of the neurite tracing points to obtain a tracing with a uniform neurite radius of value 1.0 (for example, for getting a representation where branching patterns can be appreciated more clearly, without the influence of neurite width). To achieve this, the user applies a special-purpose custom correction method that sets the radius of each tracing point to 1.0. Figure 14 shows the resultant neuron morphology tracing and its corresponding reconstructed mesh.

Finally, the corrected tracings and their correspondent neuron mesh reconstructions are ready to be used for studies or comparisons. Both the neuron morphology tracings and the meshes can be saved with all the applied changes. The tracings are saved in “.swc” format and the meshes, in “.obj” format.

## 4 Discussion, conclusions, and future work

The robust acquisition of the anatomy of neuronal cells is a demanding task but an essential step for many subsequent studies about the morphology, physiology, or connectivity of neurons. Also, counting with accurate morphological data of real neurons from specific populations is relevant in computational neuroscience and other fields, such as neuro-inspired technologies, within the areas of robotics, control, and computing (including neuromorphic systems).

Morphological tracings are a common representation that is useful for describing neuron anatomy, serving as input to many of the areas mentioned above. They can be extracted from microscope images either manually or through automatic or semi-automatic techniques, and they are frequently stored in neuron repositories. However, we cannot overlook that raw tracings often require correction and refinement stages, either to remove acquisition errors or to adapt their features to the requirements of specific application areas. This demands the execution of procedures that are often error-prone and time consuming, usually requiring experienced operators who have to make context-dependent decisions often based on subjective judgment.

The tool presented here is meant to be used after a first neuron reconstruction has been made from the stack of images. Some other tools also allow postprocessing, such as Filament editor (Dercksen et al., 2013) which allows simultaneous visualization of complex neuronal tracings and image data in a 3D viewer and interconnection across sections, or Vaa3d (Peng, 2014) that allows pruning, calculation of morphological features of a reconstructed neuron, and which also has the very interesting feature of providing a reliability measure of reconstructed segments/nodes based on the calculation of alternative pathways. However, to our knowledge, NeuroEditor^1^ provides some unique characteristics. First, it has been specifically designed to facilitate and accelerate the processes of visualization, analysis, and correction of neuronal morphological tracings. It allows the inclusion of previously unacquired information or even the estimation of elements that may not be present in the original acquired tracings, such as the description of the soma or the thickness of the dendritic arbor. This may be important, for example, for incorporating neurons acquired from a repository to a multi-compartment simulation. Regarding the soma generation, the tool follows the method proposed in Brito et al. (2013) and Garcia-Cantero et al. (2017). However, in NeuroEditor, both the points used as a basis in the method to generate the soma and the generated soma itself can be edited, which allows modification of the shape of the soma and its attachment to the first-order dendrites. In addition, although we tend to obtain the morphology as detailed as possible, we sometimes run into tools that generate tracings that have a large number of irrelevant tracing points. In these cases, if desired, NeuroEditor offers the possibility of compacting and simplifying the neuron *ad hoc*. On the other hand, the tool supports large-scale reconstructed neurons. Besides, NeuroEditor provides an intuitive framework for the interactive visualization of morphological tracings, allowing the simultaneous rendering of the original and edited/corrected tracings, displaying them either side-by-side or superimposed. This option facilitates comparing the original and edited tracings, allowing the assessment of the modifications introduced on them. Additionally, a 3D mesh approximating the neuronal surface can be generated on-the-fly. This capability, not available in other tools so far, allows visualizing the effect of the editing operations instantly, not only over the tracing, but also over the neuronal membrane. Furthermore, NeuroEditor can provide accurate approximations of the soma shape, computed from the neurite distribution across its surface.

NeuroEditor offers strategies for automatic error detection and visual evaluation. On the one hand, users can get a list of nodes that are likely to be erroneous and explore them with a simple click that will focus the view on that particular node, informing what type of error it might be for its manual correction. Alternatively, the tool can automatically suggest and perform corrections of typical tracing errors, as well as perform noncritical operations such as resampling or smoothing the morphological tracings. Methods for automatic error detection can be selected from the list of procedures supplied with NeuroEditor, or can be specifically developed by the user, by incorporating Python code to adapt the tool to the user’s specific needs. This is a very relevant feature since the criteria applied for error identification and the correction actions can vary widely under different circumstances. However, the automatic error correction and detection features are intended to be used on whole neurons, or at least whole neuritic branches, and unexpected results may be obtained for multiple orphan segments or isolated branches. Users can add their own Python scripts following a given structure in a simple way, without needing neither to know the architecture of the application nor to recompile it. The combination of all these options allows the flexible configuration of processing pipelines that associate the automatic identification of errors with the execution of correction actions, which may involve either the execution of automatic correction methods or the generation of warnings so that users can explore them and, guided by the tool, decide on the most appropriate action in each case.

The development of tools for facilitating this post processing task such as NeuroEditor is central for freeing skilled operators from tedious work, reducing the review time and improving the quality of the acquired tracings. In addition, the presented application includes the generation of geometric models of the surface of the neuronal membrane, reconstructed from the improved and homogenized tracings. These three-dimensional generated models can be then used in the functional simulation of neurons, the development of neuro-inspired systems or the computation of morphological properties that are not possible to obtain directly from the tracings.

As future work, we plan to include the visualization of the original microscopy images, providing therefore a complete view of the whole reconstruction process: The initial microscopy data, the original tracing, the results of the tracing editing process and the neuronal membrane reconstructed from the tracing and superimposed over the microscopy image. The set of images generated this way would provide a useful combination of information for assessing the results of the tracing and editing procedures. Additionally, it would be useful to count with tools that allow to quantify the differences between two neurons. This would allow us to provide quantitative measures about the resulting neuron when correcting errors or simplifying tracings.

Further work also includes the extension of the editing and reconstruction operations to other filiform morphological structures that suffer from similar problems, such as the brain vascular system, whose description is obtained similarly to the neuron morphology tracings.

## Data availability statement

The original contributions presented in the study are included in the article/Supplementary material, further inquiries can be directed to the corresponding author. The code developed during this research can be found in https://github.com/vg-lab/NeuroEditor and in https://vg-lab.es/neuroEditor/ can be found an executable for Windows 7, Ubuntu 16.04 and MacOs as well as a manual and an explanatory video.

## Ethics statement

Ethical approval was not required for the study involving humans in accordance with the local legislation and institutional requirements. Written informed consent to participate in this study was not required from the participants or the participants’ legal guardians/next of kin in accordance with the national legislation and the institutional requirements.

## Author contributions

IV: Software, Visualization, Writing – review & editing. JG-C: Conceptualization, Visualization, Writing – original draft, Data curation, Formal analysis, Investigation, Methodology. JB: Conceptualization, Writing – original draft, Writing – review & editing. SB: Writing – original draft, Writing – review & editing, Conceptualization, Visualization. LP: Writing – original draft, Writing – review & editing, Methodology. SM: Conceptualization, Visualization, Writing – original draft, Writing – review & editing.

## Footnotes

^1^https://vg-lab.es/neuroEditor
