# Supplementary material for: NeuroEditor: a tool to edit and visualize neuronal morphologies
Source: Front Neuroanat. 2024 Feb 14;18:1342762. doi: 10.3389/fnana.2024.1342762 (PMC10902916; doi:10.3389/fnana.2024.1342762)
Supplement: Supplementary file 1 [file Presentation_1.pdf]

Neuro Editor

User Manual

Velasco González, I., Garcia-Cantero, J. J., Brito, J. P., Bayona, S., Pastor, L., & Mata, S.

(Velasco González et al., 2024)

(VG-LAB, 2024.)

NeuroEditor

1. Main Visualization Window: 2

2. View Panel 2

3. Select Panel 4

4. Edit panel 5

   4.1. Translation & Rotation 6

   4.2. Simplification & Refinement 7

5. Correct panel 9

NeuroEditor is a software tool for the visualization and edition of morphological tracings that offers manual edition capabilities together with a set of algorithms that can automatically identify potential errors in the tracings and, in some cases, propose a set of actions in order to automatically correct them. Neuroeditor can simultaneously visualize the original tracing, the modified tracing, and a 3D mesh that approximates the neuronal membrane.

The interface (see Figure 1: Overview) is composed of a main visualization window (C), and 4 panels (**View** (A), **Select** (B) **Edit** (D) and **Simplification & Refinement** (E)) that allow the interaction with the tool.

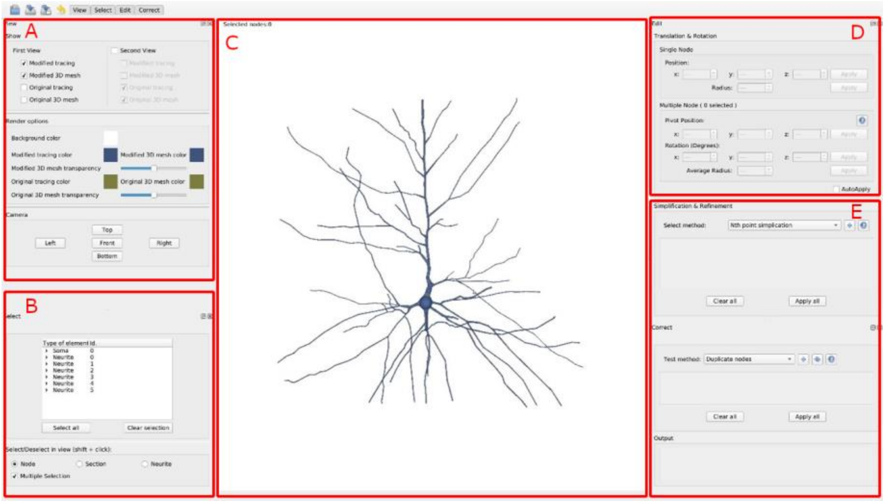

Figure 1: Overview of the application interface. The interface shows a main display panel (C) and 4 panels to access and parameterize the application functionalities: ***View*** *(A),* ***Select*** *(B),* ***Edit*** *(D) and* ***Simplification & Refinement*** *(E).*

1. Main Visualization Window:

This window shows the tracing (represented by lines and spheres) and an approximation of the dendritic membrane that is generated in real time. The visualization can be configured to select the elements to be simultaneously depicted among the following options: **original tracing**, **modified tracing**, cell membrane approximated from the original tracing (**original 3D mesh**), and cell membrane approximated from the modified tracing (**modified 3D mesh**). These options will be detailed in the next section. The events accepted in the visualization panel are listed in the following table:

Action	Shortcut	Notes
**Zoom**	Mouse Wheel	Allows to zoom in or out the camera.
**Translate camera**	Right Button (Mouse) + drag	Moves the camera through the scene
**Rotate camera**	Left Button (Mouse) + drag	Rotates the scene
**Selection/Deselection**	Shift + Right	Allows to select and deselect nodes, sections, or dendrites (this behavior can be changed in the **Selection Panel**).
Translate the selected nodes	Ctrl + Right	Translates the selected nodes
Rotate the selected nodes	Ctrl + Left	Rotates the selected nodes (no effect if only 1 node is selected)
Delete the selected nodes	R	Deletes the selected nodes
Undo	Ctrl + Z	Undoes the last action performed
Show menu	Left Button	Shows context menu with some additional options

2. View Panel

This panel (see Figure 2) allows customizing the main display and is divided into 3 areas:

- **Show**: Modifies what is to be displayed in the visualization window by allowing a side-by-side layout (see Figure 3), where each of the views is controlled independently. The available options are:
  - **Modified tracing**: when checked, the modified tracing becomes visible.
  - **Modified 3D mesh**: when checked, the membrane of the modified tracing becomes visible.
  - **Original tracing**: when checked, the original tracing becomes visible.
  - **Original 3D mesh**: when checked, the membrane of the original tracing becomes visible.
- **Render options**: This section allows customizing the colors and transparency of the displayed tracings.
- **Camera**: The camera is placed at the selected position.

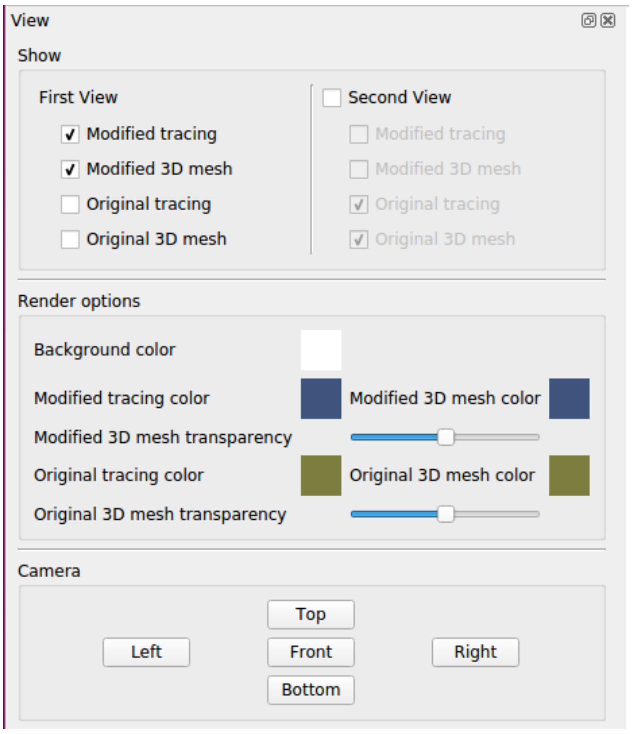

*Figure 2: The **View panel** is split into three main areas: **Show** section allows to control the displayed representations and controls the side-by-side layout; **Render options** allows to customize the colors and the transparency of the displayed representations, and **Camera** section allows the camera to be placed in predetermined locations.*

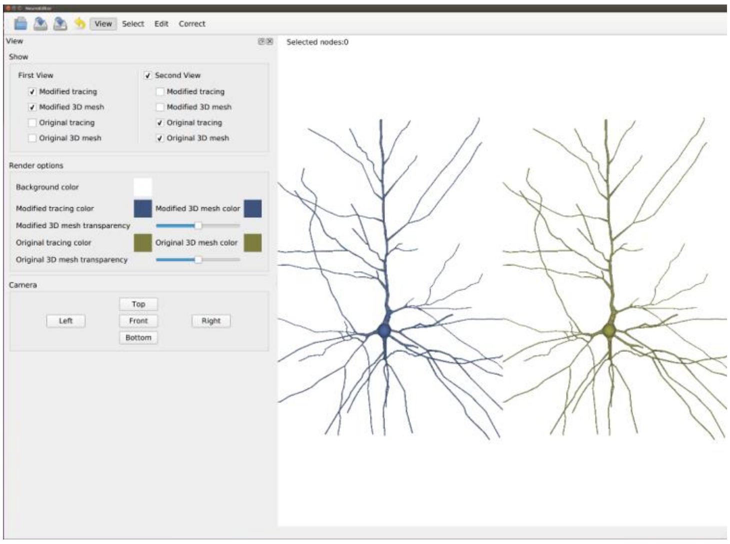

*Figure 3: In this figure, we can see an example of the side-by-side layout. The left side shows the modified tracing and membrane while the right side shows the original tracing and membrane. Note that this is configurable through the checkboxes available in the **Show** area.*

3. Select Panel

This panel (see Figure 4) is divided into two main areas, the first area provides a textual view of the hierarchical organization of the tracing (Figure 4A): The cell is formed by the Soma and one or more Neurites; each one of these Neurites is composed by Sections (portions running between two bifurcations) which, in turn, are made up of Nodes.

This hierarchical representation of the neuron provides an alternative selection environment and it is completely coordinated with the visual representation, therefore, the selections made in the tree will be reflected in the visualization and vice versa. In addition, the tree representation reflects the selection state following a color based coding: a complete selection is indicated with a blue background, (for example, “Section 32” in Figure 4A), meaning that the whole entity (and all its children, if any) is selected. A partial selection, on the other hand, is marked with blue text, for example, "Neurite 4", indicating that some of its children are selected (but not all of them).

The other area (see Figure 4B) allows the user to configure the behavior of the selection in the **main** visualization. Selections can be made individually or accumulatively (**multiple selection checkbox).** In the first case, the selection of an element cancels the selection of any other previously selected element. In the second case, the selection of an element adds up and keeps the previously selected elements; this behaviour is achieved by ticking the **Multiple selection** option. In addition, you can also configure the entities to be selected by choosing between selecting single **nodes**, **sections** or complete

neurites. For example, in case of having selected the **neurite** option in the **Select/Deselect** area, clicking on a node will select the complete neurite to which the node belongs.

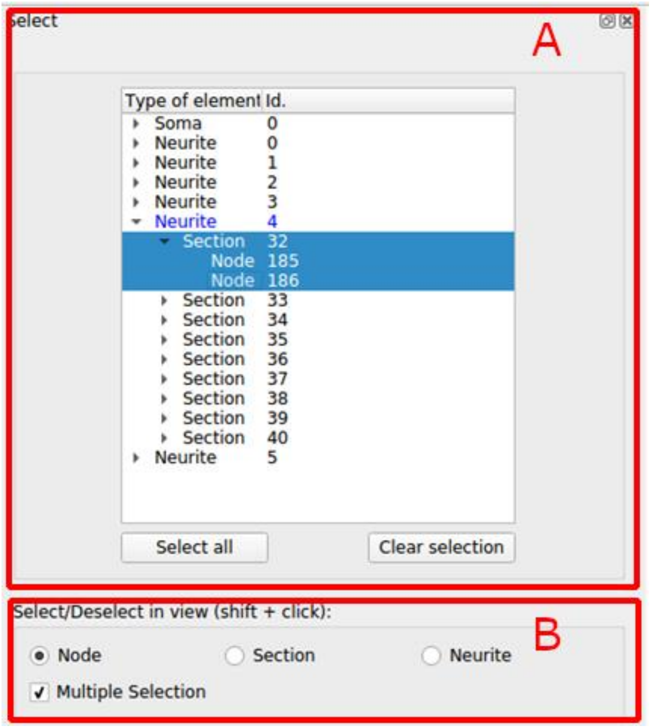

*Figure 4: The **Select Panel** is divided into two areas: the hierarchical representation (A) and the configuration of the selection behavior (B). In this example, the colors in the hierarchical representation mean that the neurite with id 4 is partially selected (the text is colored), while section 32 is completely selected (the background is colored).*

4. Edit panel

This panel (see Figure 6) allows the user to modify the tracing algorithmically, instead of editing it through mouse interaction in the visual representation. It is divided into two main areas: the **Translation & Rotation** area (see Figure 6A), which allows modifying the position of the selected nodes in a manual way, and the **Simplification & Refinement** (see Figure 6B) area, which allows applying various automatic processes to simplify or enhance the tracing.

Comentado [SF1]: Si has puesto la primera área en negrita, esta debería ir también en negrita

Comentado [SB2R1]: Esto es lo que digo, o usas negrita, o comillas, pero en todo el documento y pies de figura igual.

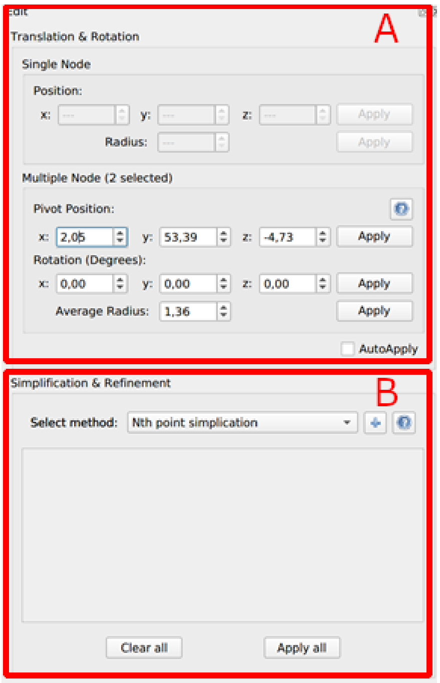

*Figure 5: The **Edit panel** is separated in two areas: **Translation & Rotation** (A) allows to translate and rotate the selected node (or nodes) and **Simplification & Refinement** (B) that allows to apply simplification and enhancement methods automatically.*

Comentado [SB3]: En todo el manual, pon coherente lo de con/sin comillas y con/sin negrita, cuando esté escrito así tal cual en la interfaz.

4.1. Translation & Rotation

This section allows to modify the position of the nodes that compose the tracing and is divided into two zones:

- **Single Node**: This zone is active while only one node is selected and the data of the node (i.e its **position** and **radius**) are displayed and can be modified.
- **Multiple Node**: This zone is active while more than one node are selected (see Figure 6). In this case, instead of displaying the position of the nodes, the average of the positions of all the selected nodes (**Pivot Position**) is displayed. Similarly, the average radius is selected and can be modified (**Average Radius).** In this case, all the selected nodes will undergo the same modification as the average radius. For example, if we start from an average radius of 0.25 and modify it to 0.20, all nodes will see their radius reduced by 0.05**.** Regarding the **Rotation** of the selected nodes, they rotate around the Pivot point, which is represented by showing the coordinate axes at the Pivot position.

Comentado [SB4]: aquí sería más correcto decir "Multiple Nodes", pero como implica un cambio en la interfaz, podría quedarse así

Finally, any modifications in the values of a node (or nodes) can be immediately observed in the visualization in case the **AutoApply** option is checked. If this option is disabled, the user should click

on the Apply buttons to transfer the new values and see the changes in the visualization. If performance issues arise, it is advisable to disable the **AutoApply** option.

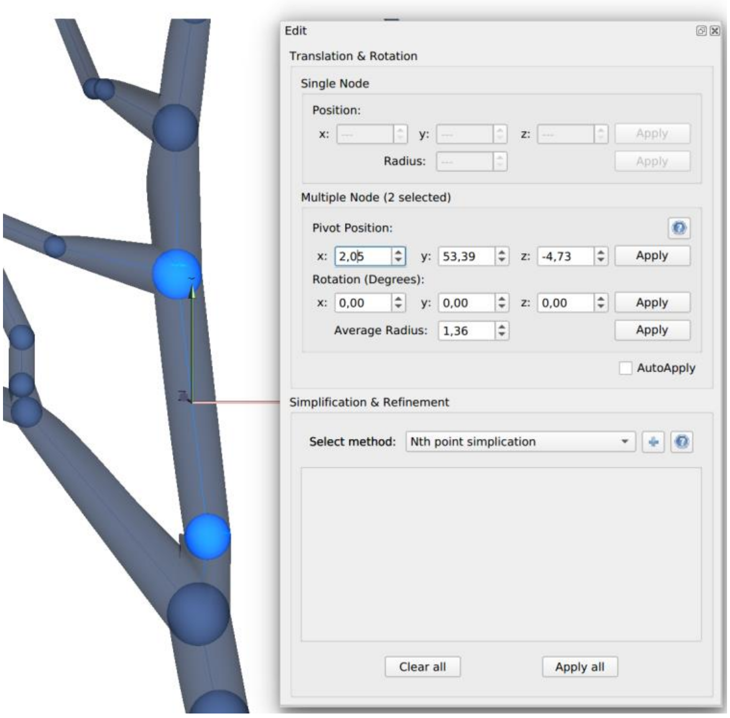

*Figure 6: Example of multiple selection in the **Edit panel**. Note that the coordinate axis represents the position of the pivot, which is the point around which the nodes are rotated.*

4.2. Simplification & Refinement

This section allows applying simplification and enhancement methods automatically. To do that, the desired methods are selected in the drop-down list and added one by one by pressing the **+** button. Once the desired methods have been added, their parameters can be configured, and the user can select to apply the methods one by one or to apply them all at once (see Figure 7). The available methods and a description of each one can be found by pressing the help button (**?**) as shown in Figure 8.

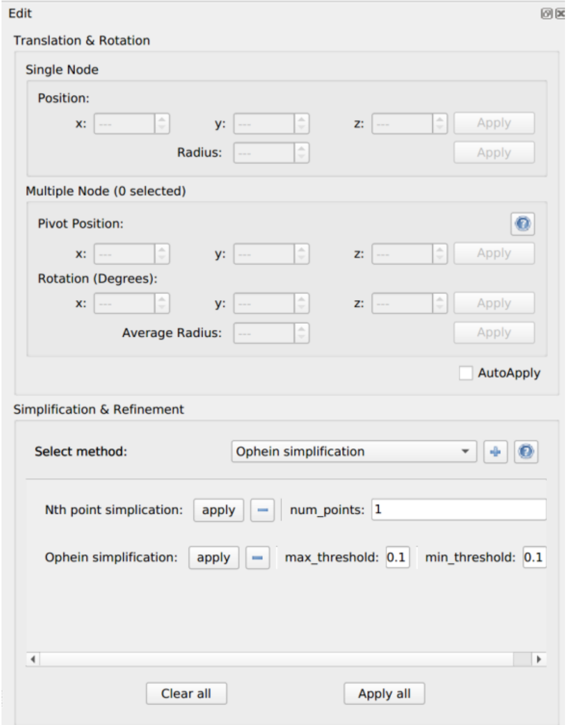

*Figure 7: In this example, two simplification methods have been selected for illustration purposes. As it can be seen, each of them has its own parameters and can be applied either individually (***Apply***) or all together following the sequence in which they were selected (***Apply All***)*

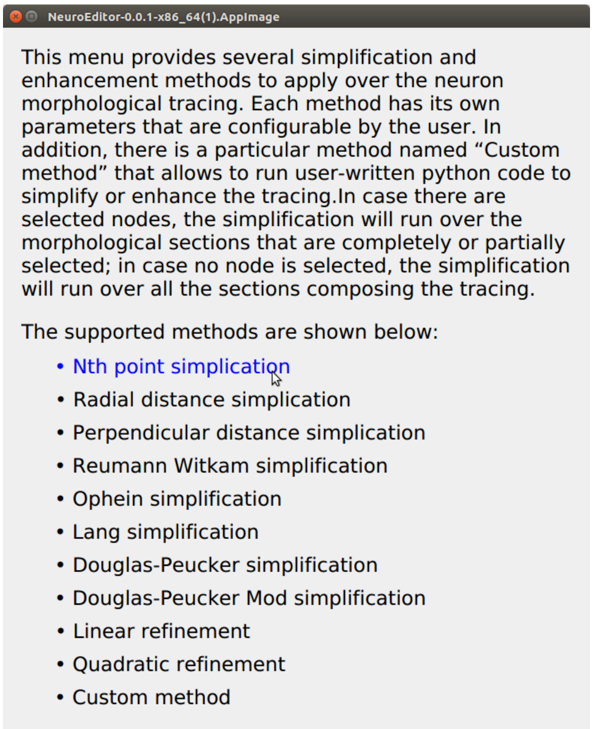

Figure 8: All available simplification and refinement methods. Note that the user can click the method name to obtain a detailed description.

5. Correct panel

This panel allows applying certain tests that detect common errors in the tracing. Then, the user can choose either to apply an automatic solution to all the errors found by the test or to manually check all the nodes presenting errors.

To add a test to be performed, select it from the drop-down list and click on the **+** button, while the **++** button adds all available tests. Once all the desired tests have been added the user can configure their behavior when an error is found. By default, all tests report the errors in a console for later review, and for each test, there are a series of default options to repair the errors found without further intervention.

In case of selecting the option to display the test results in a console, the user can edit each error individually (see Figure 9). The erroneous nodes will be displayed as buttons so that, when clicking on them, the visualization will automatically select and display the erroneous node. Besides, a drop-down menu will also be displayed to show the default correction options, although the user can always choose to manually edit the nodes in the visualization or in the edit panel. In addition, to indicate that a node has been modified after the test process (see Figure 10) (and therefore, the error may no longer be present), the button will be colored in green, this action occurs both when using the default options and with the manual editing.

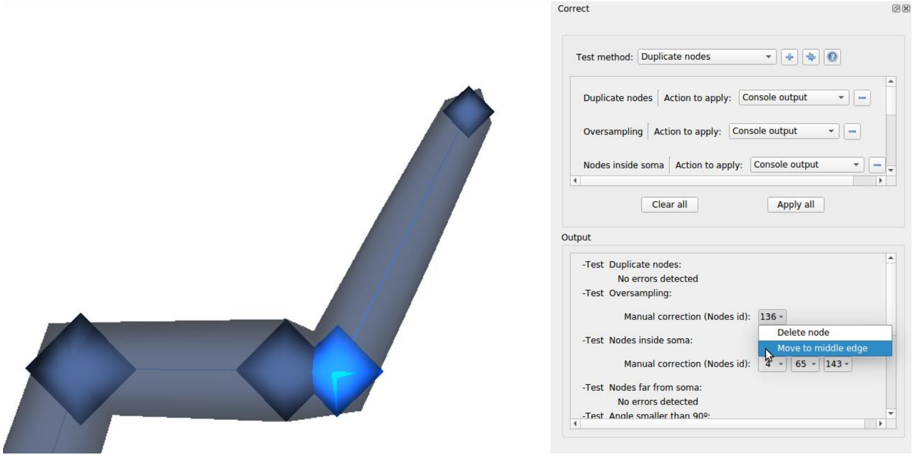

Figure 9: The node 136 is very close to another node, clicking on its corresponding button we can see in the viewer the node in question and in the drop-down menu the default options to solve the error.

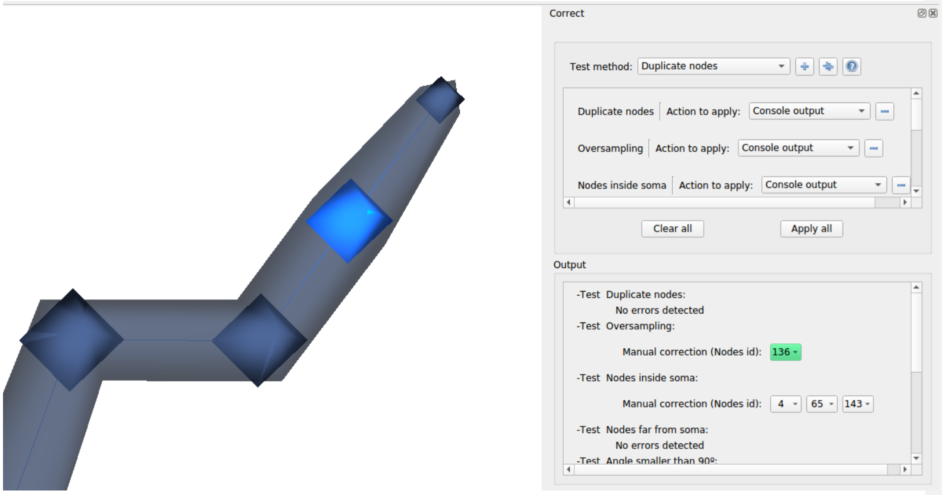

*Figure 10: Result of applying the **move to middle edge** correction to the node in the previous figure. Note how, when the node is modified, the button in the output console has changed color to indicate this modification.*

6. Bibliography

NeuroEditor (2024). Available at: https://vg-lab.es/neuroEditor/ (Accessed January 29, 2024).

Velasco González, I., Garcia-Cantero, J. J., Brito, J. P., Bayona, S., Pastor, L., and Mata, S. (2024). NeuroEditor: A tool to edit and visualize neuronal morphologies. *Front Neuroanat* 18, 1342762. doi: 10.3389/FNANA.2024.1342762
